# Treatment of Diarrhœa by Sulphuric Acid

**Published:** 1854-05

**Authors:** F. M. Davidson

**Affiliations:** Barking, Essex


From the London Lancet.

*Treatment of Diarrhœa by Sulphuric Acid.*

To the Editor of the LANCET.

SIR,—Having seen in the Lancet sulphuric acid so strongly and frequently recommended as a remedy for diarrhœa, I determined (as I had considerable opportunities of testing its efficacy) to give it a fair trial. I have now before me notes of fifty-two cases treated successfully with it. I administer the sulphuric acid in the following form:

Dilute sulphuric acid, two drachms; simple syrup, two drachms; tincture of opium, forty minims; water, six ounces. The patient to take a sixth part of the above mixture after each loose motion; in many cases two doses of the mixture are sufficient to check the diarrhœa. If the patient complains of cramp and pain in the abdomen, I order hot turpentine fomentations, and half or a third of a grain of morphia. If the vomiting is troublesome, a liberal supply of ice is allowed, which in this town is easily procured.

Sulphuric acid, as a remedy in diarrhœa, has many and great advantages over the chalk mixture. The latter is exceedingly nauseous, but the sulphuric acid is so pleasant that it is readily taken by children. It is decidedly more efficacious; it allays the thirst which accompanies diarrhœa; it is not precipitated; it is not afterwards followed by the troublesome constipation which attends the use of chalk mixture, and it is a very cheap remedy. Of course I do not administer the sulphuric acid in those cases o diarrhœa arising from the presence of irritating or non-digested food in the stomach or alimentary canal.

Cholera (in all probability) will soon be here, and others, like myself may perhaps be induced to try this truly valuable remedy in combating its premonitory diarrhœa.

I remain, Sir, your obedient servant,

F. M. DAVIDSON.

Barking, Essex, March, 1854.

